# Supplementary material for: Targeting the HIV-infected brain to improve ischemic stroke outcome
Source: Nat Commun. 2019 May 1;10:2009. doi: 10.1038/s41467-019-10046-x (PMC6494822; doi:10.1038/s41467-019-10046-x)
Supplement: Supplementary file 1 — Supplementary Information [file 41467_2019_10046_MOESM1_ESM.pdf]

Supplementary Information

Targeting the HIV-infected brain to improve ischemic stroke outcome

Luc Bertrand*, Fannie Méroth, Marie Tournebize, Ana Leda, Enze Sun and Michal Toborek*

University of Miami Miller School of Medicine, Department of Biochemistry and Molecular Biology, Miami, FL

Corresponding author:

Michal Toborek (mtoborek@med.miami.edu) or Luc Bertrand (l.bertrand@med.miami.edu) University of Miami Miller School of Medicine, Department of Biochemistry and Molecular Biology, 1011 NW 15th Street, Miami, FL 33136, USA, Phone: 305-243-0230, Fax: 305-243-3955

Supplementary Information

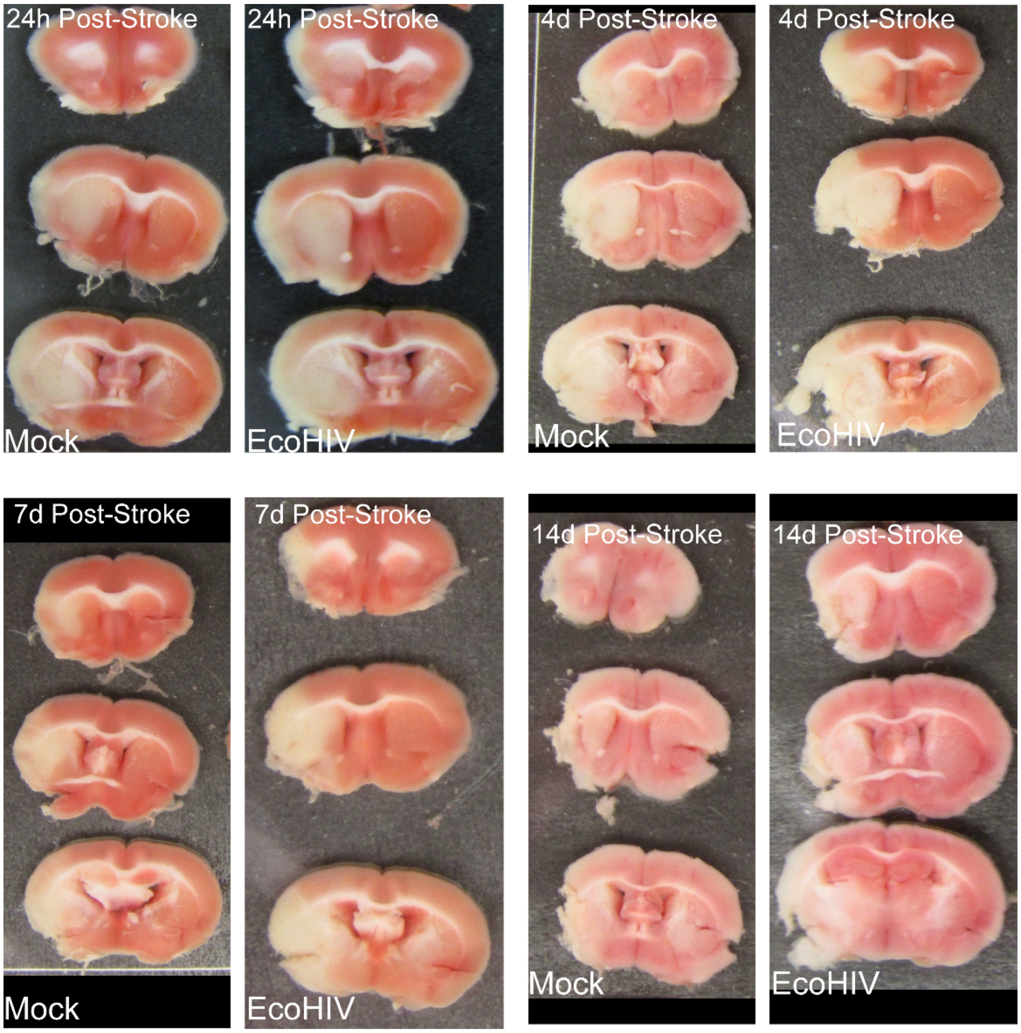

<u>Supplementary Figure 1. Post-ischemic stroke brain lesions as evaluated by TTC staining.</u> Representative images for each group and time point presented in Fig 1. Three 1 mm thick sections are shown, infarct area is visible by the absence of red TTC staining.

Supplementary Information

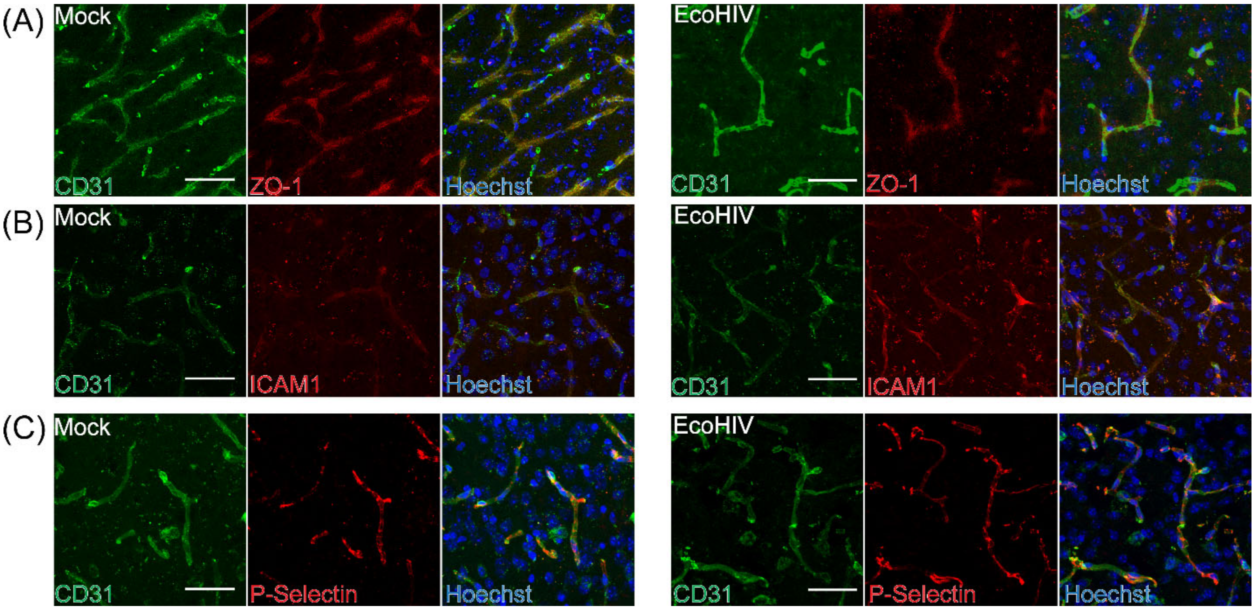

Supplementary Figure 2. Microvessel visualization and quantification of ZO-1, ICAM-1 and P-selectin. Related to Figs. 2 and 3. Representative three panel presentation of the triple-stained brain sections for CD31 (green) and ZO-1 (A), ICAM1 (B), and P-Selectin (C) (all on red). Blue represent Hoechst nuclear staining. Left panels represent Mock-treated and right panels represent EcoHIV-infected mice. (A-C) Scale bars 40 µm.

Supplementary Information

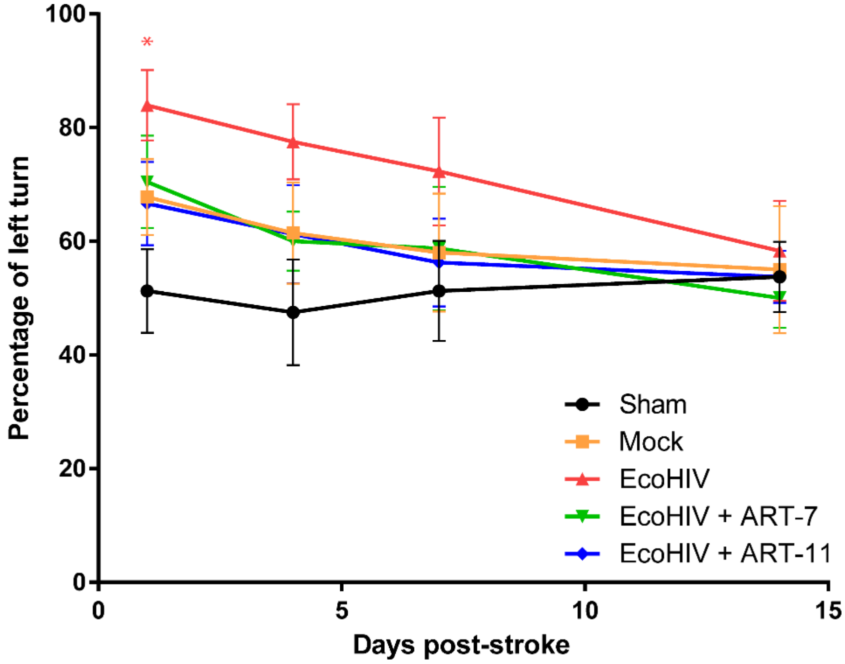

Supplementary Figure 3. Sensorimotor evaluation. Related to Fig 8. Mice were infected, treated with ART-7 and ART-11, and subjected to stroke as in Figs 6 and 7, followed by evaluation for sensorimotor deficit using the corner test at days 1, 4, 7, and 14 post-ischemic stroke; n=9-20 per group, 8 assays per mice per time point, 2 independent experiments. Data presented as mean and SEM. Source data are provided as a Source Data file.*p<0.05 Sham vs EcoHIV. One-way ANOVA, followed by Tukey multiple comparison test.
