# Supplementary material for: Targeting the HIV-infected brain to improve ischemic stroke outcome
Source: Nat Commun. 2019 May 1;10:2009. doi: 10.1038/s41467-019-10046-x (PMC6494822; doi:10.1038/s41467-019-10046-x)
Supplement: Supplementary file 3 — Reporting Summary [file 41467_2019_10046_MOESM3_ESM.pdf]

nature research

Corresponding author(s): Michal Toborek

Last updated by author(s): Mar 27, 2019

Reporting Summary

Nature Research wishes to improve the reproducibility of the work that we publish. This form provides structure for consistency and transparency in reporting. For further information on Nature Research policies, see Authors & Referees and the Editorial Policy Checklist.

Statistics

For all statistical analyses, confirm that the following items are present in the figure legend, table legend, main text, or Methods section.

n/a	Confirmed	
☐	☒	The exact sample size (*n*) for each experimental group/condition, given as a discrete number and unit of measurement
☐	☒	A statement on whether measurements were taken from distinct samples or whether the same sample was measured repeatedly
☐	☒	The statistical test(s) used AND whether they are one- or two-sided<br>*Only common tests should be described solely by name; describe more complex techniques in the Methods section.*
☒	☐	A description of all covariates tested
☒	☐	A description of any assumptions or corrections, such as tests of normality and adjustment for multiple comparisons
☒	☐	A full description of the statistical parameters including central tendency (e.g. means) or other basic estimates (e.g. regression coefficient) AND variation (e.g. standard deviation) or associated estimates of uncertainty (e.g. confidence intervals)
☒	☐	For null hypothesis testing, the test statistic (e.g. *F*, *t*, *r*) with confidence intervals, effect sizes, degrees of freedom and *P* value noted<br>*Give P values as exact values whenever suitable.*
☒	☐	For Bayesian analysis, information on the choice of priors and Markov chain Monte Carlo settings
☒	☐	For hierarchical and complex designs, identification of the appropriate level for tests and full reporting of outcomes
☒	☐	Estimates of effect sizes (e.g. Cohen's *d*, Pearson's *r*), indicating how they were calculated

Our web collection on statistics for biologists contains articles on many of the points above.

Software and code

Policy information about availability of computer code

Data collection	*Provide a description of all commercial, open source and custom code used to collect the data in this study, specifying the version used OR state that no software was used.*
Data analysis	*Provide a description of all commercial, open source and custom code used to analyse the data in this study, specifying the version used OR state that no software was used.*

For manuscripts utilizing custom algorithms or software that are central to the research but not yet described in published literature, software must be made available to editors/reviewers. We strongly encourage code deposition in a community repository (e.g. GitHub). See the Nature Research guidelines for submitting code & software for further information.

Data

Policy information about availability of data

All manuscripts must include a data availability statement. This statement should provide the following information, where applicable:

- Accession codes, unique identifiers, or web links for publicly available datasets
- A list of figures that have associated raw data
- A description of any restrictions on data availability

The source data underlying Figures 1 to 8 and Supplementary Figure 3 are provided as a Supplementary Source Data file. All other data supporting the findings of this manuscript are available from the corresponding authors upon reasonable request.

Field-specific reporting

Please select the one below that is the best fit for your research. If you are not sure, read the appropriate sections before making your selection.

☒ Life sciences ☐ Behavioural & social sciences ☐ Ecological, evolutionary & environmental sciences

For a reference copy of the document with all sections, see nature.com/documents/nr-reporting-summary-flat.pdf

Life sciences study design

All studies must disclose on these points even when the disclosure is negative.

Sample size	Sample sizes were extrapolated from results obtained from a previously published study from which group means and variance were established. Using a power analysis we determined that a total of 12 animals per groups would be necessary to obtain significantly different results. Experiments were conducted with 6 animals per group at the time given the complex surgical methods involved. Experiments were repeated at least twice. Sample numbers were then re-evaluated (Significance: No repeats; Close to significance: Repeat; No change observed: No repeats.)
Data exclusions	Some samples were omitted on the basis of a failed surgery. The MCAO technique has about 85-90% success rate; if an animal did not develop ischemic-stroke, it was removed from the study. No other exclusion was performed.
Replication	Experiment were replicated at least twice, usually three times. All presented data is from independent replicates.
Randomization	Animal allocation to experimental groups was done randomly.
Blinding	Behavior and neurodeficit evaluation was performed using numbered cages with no indication of study groups.

Reporting for specific materials, systems and methods

We require information from authors about some types of materials, experimental systems and methods used in many studies. Here, indicate whether each material, system or method listed is relevant to your study. If you are not sure if a list item applies to your research, read the appropriate section before selecting a response.

Materials & experimental systems

n/a	Involved in the study	
☐	☒	Antibodies
☒	☐	Eukaryotic cell lines
☒	☐	Palaeontology
☐	☒	Animals and other organisms
☒	☐	Human research participants
☒	☐	Clinical data

Methods

n/a	Involved in the study	
☒	☐	ChIP-seq
☒	☐	Flow cytometry
☒	☐	MRI-based neuroimaging

Antibodies

Antibodies used

Western Blot:
Iba1 (Abcam ab17884, 1:1000),
GFAP (Cell Signaling 12389, 1:1000),
ICAM1 (Abcam ab179707, 1:750),
GAPDH (Novus Biologicals NB600-502FR).

Immunofluoresence:
laminin (Rabbit, Abcam ab11575),
ZO-1 (Rabbit, ThermoFisher61-7300),
ICAM1 (Rabbit, Abcam ab179707),
P-selectin (Mouse , Abcam ab6632),
Ly6g (Rat, Abcam ab25377),
MAP2 (Rabbit, Cell Signaling8707S; Mouse, Abcam ab11267),
p24 (Human, NIH AIDS Reagent Program 530),
Iba1 (Goat , Abcam ab5076),
GFAP (Rabbit, Cell Signaling 12389),
CD31 (Mouse, Abcam ab24590),
Tmem119 (Rabbit, Abcam ab209064)

Validation

Western Blot:
Iba1 (Abcam ab178846, 1:1000): Tested by manufacturer to work with the application and species used. In house evaluation

using positive cells lines or tissues. 20 references.
GFAP (Cell Signaling 12389, 1:1000), Tested by manufacturer to work with the application and species used. In house evaluation using positive cells lines or tissues. 27 references
ICAM1 (Abcam ab179707, 1:750), Tested by manufacturer to work with the application and species used. In house evaluation using positive cells lines or tissues. 2 references.
GAPDH (Novus Biologicals NB600-502FR). 33 references

Immunofluoresence:
laminin (Rabbit, Abcam ab11575), Tested by manufacturer to work with the application and species used. In house evaluation using positive cells lines or tissues. 245 references.
ZO-1 (Rabbit, ThermoFisher 61-7300), Tested by manufacturer to work with the application and species used. In house evaluation using positive cells lines or tissues. 684 references
ICAM1 (Rabbit, Abcam ab179707), Tested by manufacturer to work with the application and species used. In house evaluation using positive cells lines or tissues. 2 references
P-selectin (Mouse , Abcam ab6632), Tested by manufacturer to work with the application and species used. In house evaluation using positive cells lines or tissues. 7 references
Ly6g (Rat, Abcam ab25377), Tested by manufacturer to work with the application and species used. In house evaluation using positive cells lines or tissues. 51 references
MAP2 (Rabbit, Cell Signaling 8707S; Mouse, Abcam ab11267), Tested by manufacturer to work with the application and species used. In house evaluation using positive cells lines or tissues. CS: 12 references; Ab: 74 references.
p24 (Human, NIH AIDS Reagent Program 530), Hybridoma isolated antibody. This antibody reacts with HIV lysate in ELISA. It reacts with the p55 core precursor as well as p24 and several intermediates as determined by Western blot. The antibody stains the cytoplasm of HIV infected cell lines. Validated in our laboratory by comparing mock and infected cell lysates for western blot and immunofluoresence. Isolation reference: Gorny, M. K., Gianakakos, V., Sharpe, S., & Zolla-Pazner, S. (1989). Generation of human monoclonal antibodies to human immunodeficiency virus. Proc Natl Acad Sci U S A, 86(5), 1624-1628.
Iba1 (Goat , Abcam ab5076), Tested by manufacturer to work with the application and species used. In house evaluation using positive cells lines or tissues. 316 references
GFAP (Rabbit, Cell Signaling 12389), Tested by manufacturer to work with the application and species used. In house evaluation using positive cells lines or tissues.
CD31 (Mouse, Abcam ab24590), Tested by manufacturer to work with the application and species used. In house evaluation using positive cells lines or tissues. 68 references
Tmem119 (Rabbit, Abcam ab209064) Tested by manufacturer to work with the application and species used. In house evaluation using positive cells lines or tissues. 8 references

Animals and other organisms

Policy information about studies involving animals; ARRIVE guidelines recommended for reporting animal research

Laboratory animals	Mice, C57BL6J, 12 weeks old
Wild animals	*Provide details on animals observed in or captured in the field; report species, sex and age where possible. Describe how animals were caught and transported and what happened to captive animals after the study (if killed, explain why and describe method; if released, say where and when) OR state that the study did not involve wild animals.*
Field-collected samples	*For laboratory work with field-collected samples, describe all relevant parameters such as housing, maintenance, temperature, photoperiod and end-of-experiment protocol OR state that the study did not involve samples collected from the field.*
Ethics oversight	Manipulations were conducted in accordance to IACUC approved protocol (18-009). Facilities are AAALAC certified.

Note that full information on the approval of the study protocol must also be provided in the manuscript.
